# Conserved mechanisms of plant lipidome remodeling under heat and cold stresses revealed through a systematic review and meta-analysis

**DOI:** 10.1093/jxb/erag183

**Published:** 2026-04-17

**Authors:** Malarvizhi Sathasivam, Doug K Allen, Vijay Shankar, Rajib Saha, Sruthi Narayanan

**Affiliations:** Department of Plant and Environmental Sciences, Clemson University, Clemson, SC 29634, USA; Donald Danforth Plant Science Center, St. Louis, MO, USA; Center for Human Genetics, Clemson University, Greenwood, SC, USA; Department of Chemical and Biomolecular Engineering, University of Nebraska-Lincoln, Lincoln, NE, USA; Department of Plant and Environmental Sciences, Clemson University, Clemson, SC 29634, USA; Iowa State University of Science and Technology, USA

**Keywords:** Cold stress, fatty acid unsaturation, heat stress, lipid remodeling, membrane lipids, membrane stability, meta-analysis, plant lipidomics, very-long-chain fatty acids

## Abstract

Increasing temperature fluctuations threaten crop productivity worldwide, emphasizing the need for a deeper understanding of plant adaptation to such extremes. Lipids are fundamental biological molecules that furnish structural, metabolic, and regulatory roles in plant growth and development, and responses to environmental stresses. The potential of lipids as key targets for crop improvement under changing climates is emerging. This systematic review and meta-analysis presents comprehensive syntheses of current knowledge on plant lipidome responses to heat and cold stresses. The analysis reveals conserved lipidomic responses to heat and cold stresses across plant species, tissue types, and growth stages. The decreased levels of lipids with relatively smaller head groups [e.g. monogalactosyldiacylglycerol (MGDG) and phosphatidylethanolamine (PE)] that promote membrane bilayer structure, a decrease in unsaturation index in membrane lipids, and sequestration of polyunsaturated acyl chains into neutral lipids (e.g. triacylglycerols) emerged as conserved strategies for heat adaptation. Also, very-long-chain fatty acids were identified as important in heat stress adaptation, as their presence is likely to counteract excessive membrane fluidity caused by high temperature and to maintain membrane stability under heat stress. Under cold stress, the levels of membrane lipids containing polyunsaturated acyl chains were elevated, probably as an adaptive shift favoring more fluid, flexible membranes. Further, the levels of bilayer-forming lipids [e.g. digalactosyldiacylglycerol (DGDG)] increased and those of non-bilayer-forming lipids (e.g. MGDG) decreased. Overall, this article synthesizes knowledge of lipidome remodeling in plants and its role in resilience to temperature stress, identifying priority areas for future research to support climate-resilient agriculture.

## Teaser

This paper presents a comprehensive synthesis of current knowledge on plant lipidome responses to heat and cold stresses through a systematic review and meta-analysis.

## Introduction

In 2015, the United Nations launched the Sustainable Development Goals (SDGs) as a comprehensive framework of 17 interconnected aims to end poverty, protect the planet, and enhance prosperity for all by 2030 (UN, 2015). As of 2025, progress on several of these goals remains uneven, with many targets, particularly those related to climate action, food security, and environmental protection, facing significant challenges (UN, 2025). One of the most pressing threats to achieving the SDGs is climate change. Since the pre-industrial period, global surface temperatures have increased by ∼1.2 °C, with projections suggesting a rise of 2.7 °C by 2100 under current national commitments (IPCC, 2023). These rising temperatures, alongside increasing greenhouse gas emissions and habitat degradation, are driving more frequent and intense extreme weather events, such as droughts, floods, and heatwaves, which threaten agricultural productivity and global food systems (FAO, 2022).

The implications of climate change for food security are profound. Currently, ∼700 million people are undernourished, and climate-induced disruptions in crop yields, particularly in vulnerable regions, are expected to exacerbate this problem (UN, 2025). Yield declines of up to 25% are projected for major staple crops such as wheat (*Triticum aestivum* L.), maize (*Zea mays* L.), and rice (*Oryza sativa* L.) by mid-century under high-emission scenarios (Zhao *et al*., 2017). Moreover, extreme weather stress not only reduces yields but also compromises the nutritional quality of crops, including protein and micronutrient content (Myers *et al*., 2014). These trends underscore the pressing need for integrated research efforts that enhance crop resilience and sustainability through innovative approaches that bridge plant biology, biotechnology, and ecology.

Lipids are essential biological components that play a pivotal role in plant adaptation to environmental stresses. They are structurally and functionally diverse molecules that are central to cellular integrity, signaling, and energy storage. Plant lipids, including membrane lipids (e.g. phospholipids, glycolipids, and sphingolipids), neutral lipids [e.g. triacylglycerols (TGs)], and signaling lipids (e.g. oxylipins), make up a significant proportion of the metabolic output of cells and plant biomass (Dörmann and Benning, 2002; Li-Beisson *et al*., 2013). Waxes, cutin, and suberin are also lipids, which are essential for desiccation tolerance and pathogen defense. The remodeling of lipid structures under abiotic stress (e.g. heat, drought, and salinity), resulting in compositional changes, has the potential to affect membrane fluidity, signaling via reactive oxygen species, and stress resilience (Welti *et al*., 2002; Narayanan *et al*., 2018, 2020; Djanaguiraman *et al*., 2020; Zoong *et al*., 2020, 2021; Narayanan, 2021; Chen *et al*., 2023; Qian *et al*., 2023; Spivey *et al*., 2023; Kenchanmane *et al*., 2024; Zhu *et al*., 2024; Murphy *et al*., 2025; Jaroensuk *et al*., 2026). This biochemical plasticity positions lipids as key targets for crop improvement strategies to enhance environmental stress tolerance and resource efficiency.

Over the past few decades, lipidomics has made rapid progress in understanding plant metabolism under altered temperature conditions (Higashi *et al*., 2015; Narayanan *et al*., 2016a, b; Marla *et al*., 2017; Sattari Vayghan *et al*., 2020; Zhu *et al*., 2022; Leconte *et al*., 2025). The field of lipidomics has rapidly expanded to characterize lipid alterations under temperature fluctuations (Henschel *et al*., 2024). This was further anchored by advances in analytical chemistry (MS and chromatography techniques) that enabled comprehensive profiling of hundreds of lipid species, driving lipidomics as a powerful tool to elucidate plant resilience to climate-driven temperature extremes (Rustam and Reid, 2018; Li *et al*., 2024; Wang *et al*., 2025; Watson-Lazowski *et al*., 2025). Lipids play central roles in plant physiology and stress response. As a result, research on plant lipids directly supports multiple SDG priorities, including Zero Hunger (Goal 2), Good Health and Well-being (Goal 3), Affordable and Clean Energy (Goal 7), and Climate Action (Goal 13). Beyond their importance in food production, plant-derived lipids are also critical for renewable bioenergy development, bioplastics, and other bio-based products, making them integral to a sustainable bioeconomy (Carlsson *et al*., 2007; Xu and Shanklin, 2016). This paper synthesizes advances in plant lipid research with an emphasis on their role in plant adaptations to temperature stresses.

## Scope and structure of the review

This review was performed following the Collaboration for Environmental Evidence CEE (2022) systematic review protocol (Haddaway *et al*., 2017), which represents the state-of-the-art approach for conducting a systematic review by ensuring transparency and minimizing bias. The review presents a comprehensive synthesis of current knowledge on plant lipidome responses to temperature stress, underpinned by a systematic review and meta-analysis of published papers between 1974 and 2024. The scope encompasses a broad range of plant species, including model systems, crops, and wild relatives, and spans both heat and cold stresses across diverse tissues and developmental stages. The meta-analysis quantitatively evaluates lipid remodeling patterns of membrane and neutral lipids, including lesser-studied groups such as sterols and oxidized and acylated lipids, as well as lipids with very-long-chain fatty acids (VLCFAs), whose roles in stress adaptation are emerging. Finally, the review highlights critical knowledge gaps and methodological challenges, providing recommendations for standardization and future research directions. We aim to identify key opportunities to leverage lipid metabolism to develop next-generation crops better suited for a changing climate.

## Materials and methods

### Literature search

A comprehensive literature search was conducted across general, subject-specific, and gray literature databases. The resources included the Web of Science Core Collection (comprising BIOSIS Citation Index, BIOSIS Previews, ProQuest Theses and Dissertations, MEDLINE, and SCIELO) and Scopus. Field-specific databases such as AGRICOLA, PubMed, and CAB Abstracts were searched to ensure coverage of domain-relevant studies. Gray literature (research published in outlets other than externally peer-reviewed journals), which refers to dissertations and theses in this work, was accessed through the ProQuest Theses and Dissertations database. The literature search used the following search string: (‘lipid’ OR ‘phosphatid’) AND (plant OR crop OR ‘wheat’ OR ‘soybean’ OR ‘peanut’ OR ‘rice’ OR ‘corn’ OR ‘Arabidopsis’ OR ‘cotton’ OR ‘maize’ OR ‘tobacco’ OR ‘barley’ OR ‘cereal’ OR ‘legume’) AND (‘temperature stress’ OR ‘high temperature’ OR ‘heat’ OR ‘cold’ OR ‘low temperature’). All search records, screening decisions, and data extraction steps were documented following the ROSES (RepOrting standards for Systematic Evidence Syntheses) systematic review framework (Haddaway *et al*., 2018).

### Screening and eligibility criteria

All retrieved records were screened using Rayyan (Ouzzani *et al*., 2016), a web-based tool designed to facilitate the systematic review process. The PICO (Population, Intervention, Comparator, Outcome) framework was employed to define inclusion and exclusion criteria (Schardt *et al*., 2007). The PICO framework was developed as follows. Population: studies involving any plant species grown under controlled environmental conditions (regulated for temperature, water, light, and humidity) were considered. Intervention: studies included applied temperature stress treatments, such as high and low temperatures, along with respective optimum temperature (controls) for each plant species (Supplementary Table S1). Some studies included multiple levels of treatment factors, such as multiple high/low-temperature values (Supplementary Table S1). All temperature levels were compared with the corresponding control temperature within the study to derive the effect size [a quantitative measure that indicates how strongly and in which direction the treatment changed the ‘outcome’ (defined below)]. Comparator: the comparator group consisted of the optimum temperature for the plant growth (non-stressed or control) against which treatment effects (high temperature or low temperature) were evaluated. Outcome: the key outcomes of interest were changes in lipid composition at the lipid class and molecular species levels, and how these lipid changes contribute to temperature responses. Experiments were excluded if they involved non-plant organisms (e.g. animals or humans), acclimation or hardiness manipulations, analyses of seasonal variation, pre-treatment conditions, or other non-temperature stress factors.

### Data extraction and appraisal

Metadata were extracted from all included articles and comprised the following elements: article title, authors, year of publication, journal, plant species, tissue type, genotype, growth stage, type of temperature stress (high and/or low temperatures), treatment temperature, stress duration, sample size, lipid extraction method, lipid-profiling platform, data processing software, lipid classes, molecular species, and quantitative data (unit of measurement, mean values, SD, SE, and fold change). For studies that did not provide data in tabular format, but presented results only as figures, the WebPlotDigitizer (a software that extracts numerical data from images) (Rohatgi, 2021) was used to extract numerical values.

A critical appraisal score was assigned to each study based on the lipid-profiling platform as follows: (i) tandem MS/MS profiling=4; (ii) inclusion of MS or ultra-performance chromatography=3; (iii) chromatography with detection (e.g. UV, flame ionization)=2, (iv) basic lipid identification via chromatography=1; and (v) no lipid-profiling platform reported=0. A critical appraisal score was also assigned to the studies based on the sample sizes reported: (i) sample size >6 per group (strong replication)=3; (ii) sample size 4–6 per group (moderate replication)=2; (iii) sample size 2–3 per group (minimal replication)=1; and (iv) sample size not reported or unclear=0.

The tissue types used for lipid extraction were chloroplast (including chloroplast and thylakoid membranes), germinating seeds (including germinated seeds, imbibed seeds, and germinating kernel embryos), seedlings [including hypocotyls and ≤4-week-old whole seedlings (shoot and/or root)], leaves (young and mature), shoots (stem+leaves and shoot mitoplast), roots, crowns, flowers (including anthers, pollen grains, and sepals), spikes, and mature seeds. The high- and low-temperature treatments tested in the studies varied depending on plant species, growth stage, and tissue type (Supplementary Table S1). In *Arabidopsis thaliana*, the low-temperature treatment involved 5 °C (cotyledon stage), −7 °C (seedling stage), and −8 °C (on the leaf rosette of 28-day-old plants). The high-temperature treatment spanned a broader range from 27 °C to 45 °C, depending on growth stage and tissue type. Similarly, in maize, soybean (*Glycine max*), rice, wheat, peanut (*Arachis hypogaea*), sorghum (*Sorghum bicolor* (L.) Moench), canola (*Brassica napus* L.), and barley (*Hordeum vulgare* L.), the low-temperature treatment ranged from −5 °C to 18 °C, and the high-temperature treatment ranged from 30 °C to 50 °C, depending on species, growth stage, and tissue type. The high- and low-temperature treatments tested in other plant species are listed in Supplementary Table S1. In this paper, the heat and cold stress treatments were defined as follows: high day and/or night temperatures were regarded as ‘heat’ stress, whereas low day and/or night temperatures were regarded as ‘cold’ stress.

## Meta-analysis

### Overview

Studies were categorized into two treatment groups: heat stress (24–55 °C) and cold stress (−8 °C to 18 °C) for the meta-analysis. Within each treatment group, studies were further categorized by plant species, growth stages, and tissue types. The mean values and corresponding SD of the response variables were extracted from the studies. If studies reported SE, the SE was calculated using the formula, SD=SE×√*n*, where *n* is the sample size.

### Effect size calculation

The effect size (a standardized measure that allows for the combination and comparison of results across studies in a meta-analysis) was calculated for each treatment group using Hedges’ g (standardized mean difference with bias correction) (Hedges, 1981), implemented through the metafor package in R (Viechtbauer, 2010). This method incorporates the SD pooled across treatment and control within each study, computes the standardized difference between treatment and control groups and sampling variance within studies, and allowed the combination of results across studies, even if reported units for lipid levels were different (Borenstein *et al*., 2009).

### Heterogeneity and sensitivity analysis

Heterogeneity reflects variability in effect sizes not attributable to sampling error, but to biological and methodological differences among studies. A heterogeneity analysis was conducted for each lipid class separately under heat and cold stress treatments (using the metafor package in R (Borenstein *et al*., 2009; Viechtbauer, 2010). Heterogeneity metrics included Cochran’s Q statistic, *I*^2^ (the percentage of total variation due to heterogeneity), and τ^2^ (between-study variance) (DerSimonian and Laird, 1986; Higgins and Thompson, 2002; Viechtbauer, 2010). To identify whether specific studies influenced pooled effect sizes, a sensitivity analysis was conducted using the leave-one-out method. This approach iteratively removes one study at a time and recalculates the overall effect size (Viechtbauer, 2010; Viechtbauer and Cheung, 2010; Higgins *et al*., 2019). Publication bias and asymmetry in study distribution (direction and variability of effect sizes indicating bias) were evaluated using funnel plots and regression tests within heat and cold stress treatment groups. All the analyses were carried out using R version 4.5.2 (R Core Team, 2025). The R codes are available on the GitHub repository (https://github.com/msathas/Meta-analysis).

### Response variables

#### Lipid class

To compare the effect sizes across lipid classes, a Bayesian hierarchical model was applied using the brms package in R, accounting for the variation among studies, lipid-profiling platform used, plant species, tissue types, and growth stages. Normal priors (a Bayesian statistic which indicates assumed probability distribution before it is derived from observed data) with mean 0 and SD 1 [N(0,1)] were used to provide regularization and ensure stable and interpretable estimates (Gelman, 2006). The effect size estimates were extracted for each combination of temperature treatment, plant species, growth stage, and tissue type for downstream analyses across the lipid-profiling platforms. Additionally, we conducted a split-platform meta-analysis within each lipid-profiling platform separately, and the results were compared with those of the pooled model. The lipid-profiling platforms reported in the studies were: (i) spectrometry; (ii) TLC (both used densitometric quantification); (iii) GC; (iv) TLC (for separation) and GC (for quantification); (v) GC-MS; (vi) LC-MS, ultra-high performance liquid chromatography–MS (UHPLC-MS), and UPLC with quadrupole (UHPLC-Q); (vii) LC–tandem MS (LC-MS/MS), which included LC- electrospray ionization (ESI)-MS/MS, LC-ESI-QqTOF, UHPLC-MS/MS, UHPLC-ESI-MS/MS, HPLC-MS/MS, UPLC-HDMS-ESI-MS, LC-QqQ-MS, UHPLC-APCI-QTOF-MS, and UPLC-QTOF-MS; and (viii) direct infusion MS/MS (DI-MS/MS), which included ESI-MS/MS and QTOF-MS/MS. The lipid-profiling platforms that were reported in at least three studies were used for the split-platform analysis within each stress. Thus, the lipid-profiling platforms used for the split analysis were DI-MS/MS, LC-MS/MS, and LC-MS for heat stress, and DI-MS/MS, LC-MS/MS, and TLC-GC for cold stress. In the pooled and split analysis, the posterior estimates (statistics derived from the updated probability distribution that combines prior information with observed data) included posterior means and 95% credible intervals (CrIs). The tidyr package in R was used to restructure the data into formats suitable for comparative analysis (Wickham *et al*., 2024), stringr for standardizing lipid class names (Wickham, 2009), purrr for iterative processing of grouped data in treatment-wise subsets (Wickham and Henry, 2025), and tibble for tidy data frame handling (Müller and Wickham, 2023). Results were visualized using heatmaps, generated with the ggplot2 package in R (Wickham, 2016).

#### Lipid molecular species

The responses of individual molecular species to heat and cold stresses were analyzed using Bayesian hierarchical models. The effect size estimates and variances for each treatment were extracted and pooled across lipid-profiling platforms. The posterior estimates included posterior means and 95% CrIs. Additionally, a split-platform analysis (per lipid-profiling platform) was performed, and the results were compared with those of the pooled analysis. Results were visualized using bar plots, generated with the ggplot2 package in R (Wickham, 2016).

#### Very-long-chain fatty acids

In this work, VLCFAs were considered as fatty acids with ≥22 carbon atoms (Řezanka and Sigler, 2009; Kyselová *et al*., 2022). Some studies reported C20 fatty acids. However, in our work, the C20 fatty acids were considered as long-chain fatty acids (LCFAs), following Řezanka and Sigler (2009) and Kyselová *et al*. (2022). Data on VLCFAs (≥ C22) were reported by 15 studies under heat and/or cold stress conditions. The data were analyzed using a Bayesian hierarchical model to estimate the effect sizes of VLCFAs under heat and cold stresses.

## Results

### Literature search and screening

**Fig. 1.**
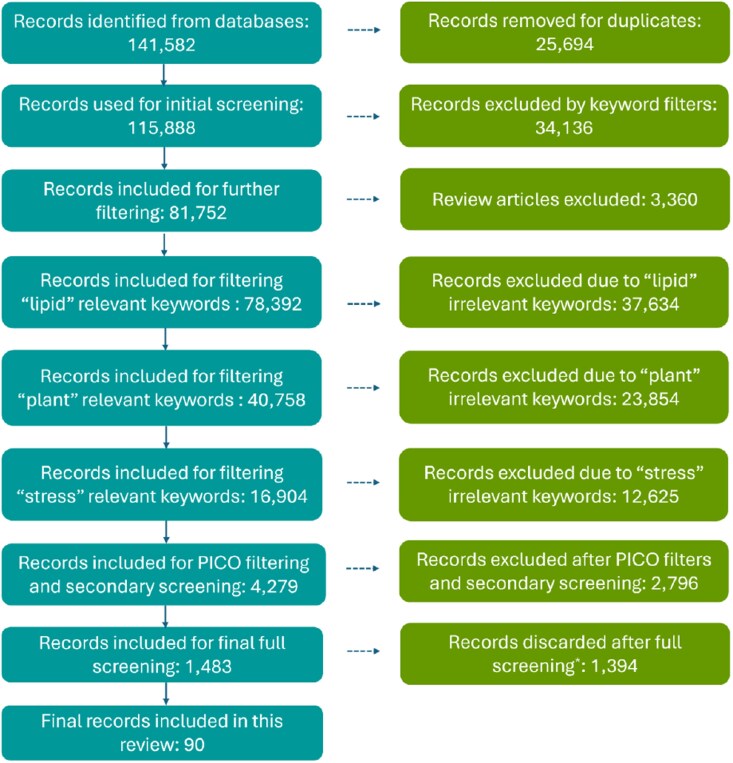
Flow diagram showing the literature screening pipeline. The left half of the flow chart (blue boxes) indicates the number of articles included in each step. The right half (green boxes) shows how articles were excluded in each step based on a specific screening criterion. The ‘lipid’-relevant keywords used for filtering the articles include lipid and phosphatid. The ‘plant’-relevant keywords include plant, crop, wheat, soybean, peanut, rice, corn, Arabidopsis, cotton, maize, tobacco, barley, cereal, and legume. The ‘stress’-relevant keywords included temperature stress, high temperature, heat, cold, and low temperature. The keyword filters for exclusion were rat, fish, fungi, bacteria, mammal, diet, mice, rodent, milk, blood, meat, murine, mouse, broiler, case study, case report, insects, larva, and survey. For a secondary screening, all the above-mentioned steps were repeated. The final full screening involved manual screening based on the inclusion keywords as mentioned above and the PICO framework (see the Materials and methods for details).

A total of 141 582 records were retrieved from the databases, which were reduced to 115 888 by Rayyan’s deduplication application (Fig. 1). The series of screening protocols involved deduplication, keyword filtering, and PICO filtering. Deduplication was performed using the auto-match option, which matches exact copies of entries, such as title, publication year, author name, and journal name. These records were subjected to keyword filtering and screened based on pre-defined inclusion and exclusion criteria. To facilitate exclusion, terms that implied lipidomics unrelated to plants were used, which removed 34 136 studies from the list. Rayyan identified 3360 review articles, which were also excluded from further consideration. For inclusion, the original search string keywords were reapplied, and the resulting articles were filtered using the PICO framework, followed by a final manual screening with full-text review to assess relevance to the scope of this systematic review. This process resulted in 90 studies being included in our systematic review (Fig. 1). The selected articles were published between 1974 and 2024, with 2020 having the highest number of publications (Supplementary Fig. S1).

### Meta-analysis

Among the studies that were used for the qualitative analysis (systematic review), those for which data were confidential or unavailable were excluded from the quantitative meta-analysis. Further, studies that did not report the SD of the variables of interest (lipids and VLCFA amounts) or reported an SE without the sample size were also excluded from the quantitative meta-analysis. Thus, out of the 90 articles included in the systematic review (qualitative analysis), only 61 articles were used for the meta-analysis (quantitative). The effect size and variance estimated for each observation in each study are given in Supplementary Table S2.

Multilevel meta-analysis models, fitted with study as the random effect, and including moderators such as temperature treatment, lipid-profiling platform, plant species, tissue type, and growth stages, significantly explained the ‘between-study’ variability (*P*<0.0001). A summary of all the tested models is provided in Supplementary Table S3. The effects of heat and cold stresses on lipid classes, lipid molecular species, and VLCFAs were examined across plant species, tissue type, and growth stages. The sensitivity analysis revealed that the pooled effect sizes remained consistent after excluding individual studies, indicating that no single study dominated the overall effect, under heat (Supplementary Fig. S2A) or cold stress (Supplementary Fig. S2B). The regression test for asymmetry using Egger’s test indicated no significant asymmetry (*z*= −1.5176, *P*=0.1291), suggesting the absence of publication bias (a funnel plot indicating the symmetry of study effect sizes is given in Supplementary Fig. S3).

### Characterization of reported data

Thirty-seven studies included in the qualitative analysis involved a minimal replication of 2–3 (critical appraisal score of one), 37 studies involved a moderate replication of 4–6 (critical appraisal score of two), and nine studies involved a strong replication of 10–20 (critical appraisal score of three). Sample size was not reported in seven studies (critical appraisal score of zero) that were published between 1974 and 2005, and those studies were retained only for qualitative analysis and not used for meta-analysis.

**Fig. 2.**
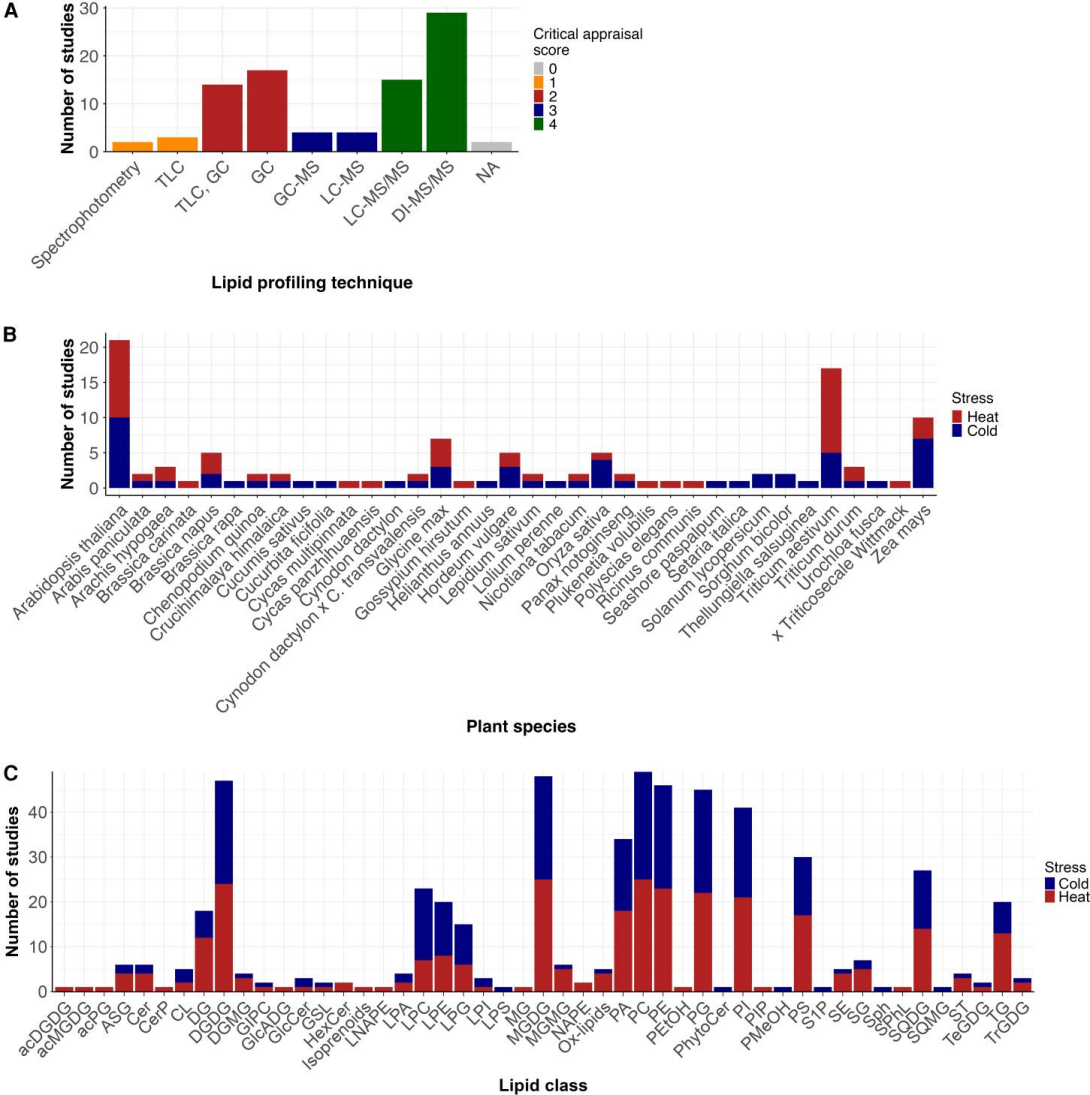
The number of studies analyzed. (A) The number of studies per lipid profiling technique (A) and per plant species (B), and the number of studies reporting various lipid classes (C). The critical appraisal score presented in (A) is a quantitative measure of the quality of methodological factors. The gray bar in (A) indicates the number of studies that did not report the lipid-profiling platforms used. (A and B) are based on the 90 studies that were used for the qualitative analysis conducted in this review; (C) is based on the 61 studies that were used for the meta-analysis. The abbreviations and expansions of lipid classes in (C) are provided in Supplementary Table S4.

Forty-four studies employed advanced lipid profiling via direct infusion MS/MS, primarily utilizing ESI-MS/MS or LC-MS/MS (critical appraisal score of four); and 44 studies employed chromatography-based (primarily GC) fatty acid analysis (critical appraisal score, 1–3) (Fig. 2A). Two studies did not report their lipid-profiling platforms (critical appraisal score of zero).

Of the 90 studies included in the qualitative analysis, 40 studies investigated plant lipid responses to heat stress, 41 studies investigated plant lipid responses to cold stress, and nine studies investigated plant lipid responses to both heat and cold stress. Of those nine studies, data collected under high temperatures were grouped under ‘heat stress’, and the data collected under low temperatures were grouped under ‘cold stress’.

Thirty-six and 23 distinct plant species were represented in the studies selected for the qualitative and quantitative analyses, respectively (Supplementary Tables S1, S2; Fig. 2B). Leaf tissues were the most studied tissue type under heat stress (33 studies) and cold stress (32 studies). Other tissue types studied under heat stress were seedlings (four studies), flowers (three studies), roots (three studies), shoots (two studies), mature seeds (two studies), chloroplasts (two studies), and germinating seeds (one study). The tissue types other than leaves studied under cold stress were roots (six studies), seedlings (five studies), crowns (three studies), shoots (three studies), germinating seeds (two studies), mature seeds (two study), chloroplasts (one study), and spikes (one study).

The most frequently reported lipid classes (in ≥25 studies) included: monogalactosyldiacylglycerol (MGDG), digalactosyldiacylglycerol (DGDG), sulfoquinovosyldiacylglycerol (SQDG), phosphatidylglycerol (PG), phosphatidylcholine (PC), phosphatidylethanolamine (PE), phosphatidylinositol (PI), phosphatidylserine (PS), and phosphatidic acid (PA) (Fig. 2C). This indicates their abundance (they are major structural lipids of plant membranes, evolutionarily conserved, and found across plant species, making them a common focus of comparative lipid studies), essential functions (indispensable for plant survival), and technical detectability (they ionize well in MS and are reproducibly detected).

### The effect of heat stress on the plant lipidome

**Fig. 3.**
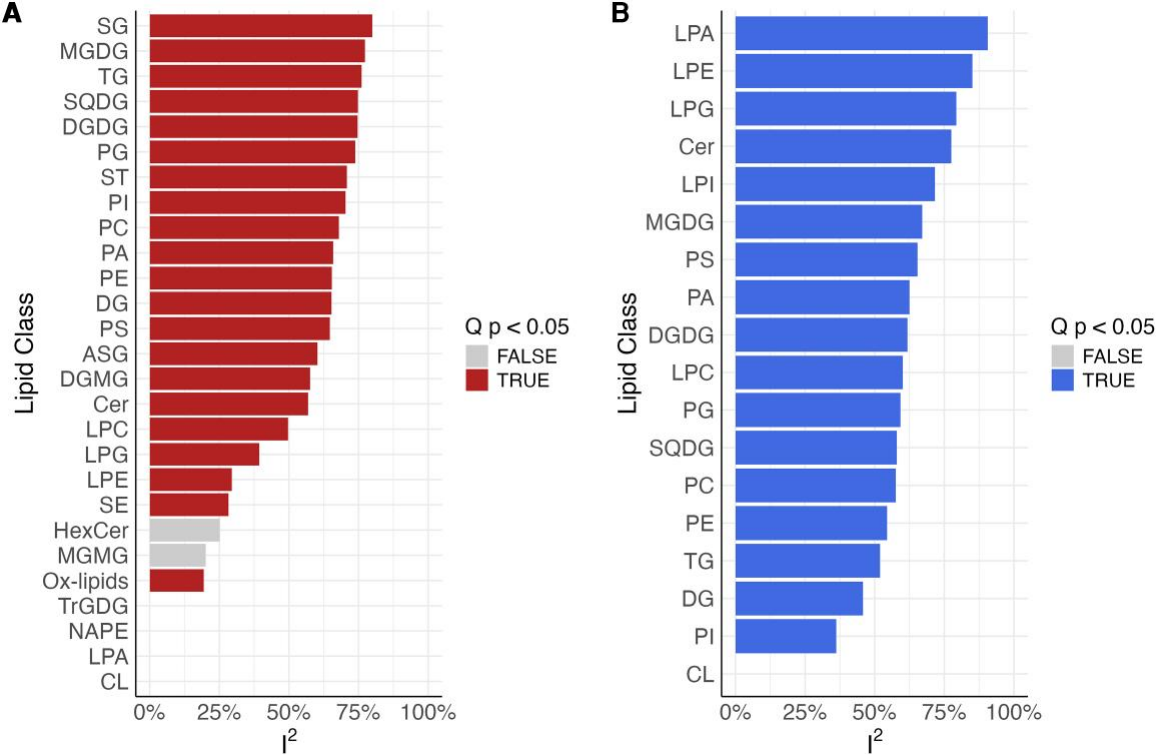
Heterogeneity analysis of lipid classes under heat and cold stress. Heterogeneity was assessed based on Cochran’s Q and *I*^2^ statistics. A significant Q-value (α, 0.05) indicates that the associated lipid class substantially varies among studies in response to heat treatment (highlighted in red in A) or cold treatment (highlighted in blue in B). The observed heterogeneity is a combined influence of biological factors (plant species, tissue, and growth stage) and technical factors (lipid-profiling platforms, sample size, and extraction protocols).

**Fig. 4.**
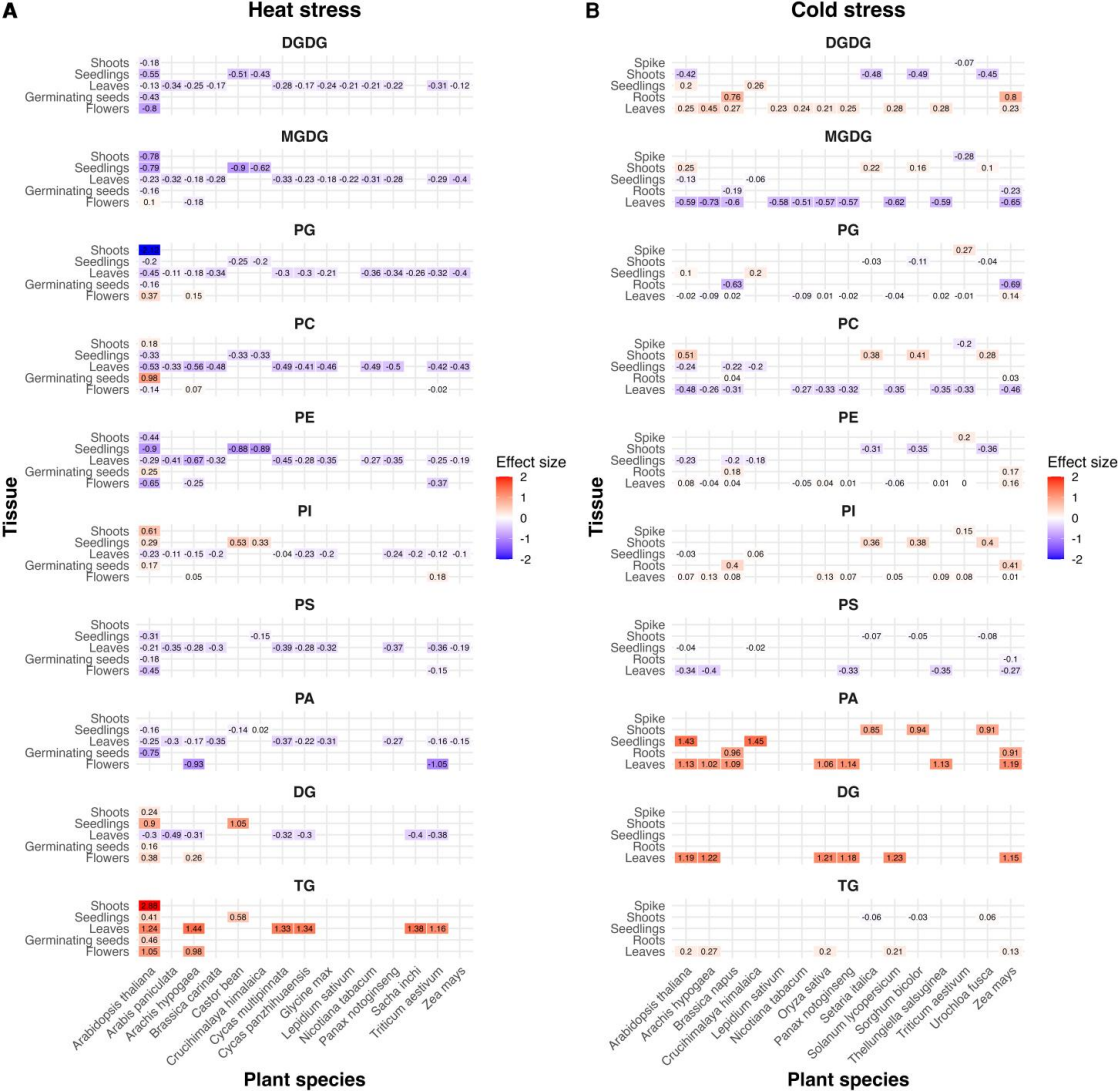
Heat map indicating the Bayesian hierarchical estimate of effect sizes for various lipid classes in different tissue types of tested plant species under strees. (A) Heat stress and (B) cold stress. Twenty-three plant species represented in the meta-analyses under heat stress and/or cold stress are presented here. The red color indicates an increase in the lipid, and the blue color indicates a decrease. Overall, this figure demonstrates the directionality of changes in lipid classes (up or down) under heat stress and cold stress.

Plants cope with heat stress by adjusting membrane composition to maintain fluidity. When the temperature increases, cells invoke lipases to remove unsaturated acyl chains in membranes that are replaced with more saturated fatty acids in an attempt to prevent the membranes from becoming too fluid, too permeable, or undergoing a phase transition to a non-bilayer phase (Upchurch, 2008; Ernst *et al*., 2016; Narayanan, 2021). Typically, the unsaturation index drops in major lipid classes such as PC, PE, MGDG, and DGDG. Evidence for reduction in unsaturation with heat comes from multiple studies (Higashi *et al*., 2015; Zoong *et al*., 2021; Sun *et al*., 2022; Spivey *et al*., 2023), and the meta-analysis here further showed consistency through an unbiased mining strategy. The analyzed plant species remodeled their lipidomes in response to heat stress (Fig. 3A; Supplementary Table S5). Based on the pooled meta-analysis conducted across lipid-profiling platforms, levels of MGDG [effect size estimate (ES) −0.48; 95% CrI −0.94 to −0.02) decreased and the levels of TG (ES 1.14; CrI 0.17 to 2.12) increased under heat stress across all plant species, tissue types, and growth stages (Supplementary Table S6). Some lipid alterations were plant species specific: for example, PE decreased in *A. thaliana* (ES −0.63; CrI −1.32 to −0.06) and peanut (ES −0.69; CrI −1.51 to −0.01) and SQDG decreased in peanut (ES −1.21; CrI −2.44 to −0.18) across the tissue types and growth stages, while the levels of the same lipid classes did not change in other species under heat stress (Supplementary Table S7). Some lipid alterations were tissue specific; for example, PA (ES −0.93; CrI −1.66 to −0.15), PG (ES −2.04; CrI −3.37 to −0.63), and SQDG (ES −2.00; CrI −3.81 to −0.14) decreased under heat stress in flowers, shoots, and germinating seeds, respectively, across plant species and growth stages (Supplementary Table S8). The detailed responses for each plant species and tissue type are presented in Fig. 4A and Supplementary Table S9.

The split-platform analysis (i.e. data were analyzed on DI-MS/MS, LC-MS/MS, and LC-MS platforms separately) revealed results that were both similar to and distinct from those of the pooled analysis. For example, LC-MS/MS detected an increase in TG (ES 1.47; CrI 0.16 to 2.66) under heat stress across all plant species, tissue types, and growth stages, similar to the pooled analysis (Supplementary Table S10). However, DI-MS/MS and LC-MS did not detect any difference in TG levels, and none of the platforms detected any changes in MGDG levels. When the plant species-specific alterations were checked within the lipid-profiling platforms, we found that DI-MS/MS detected the decrease in SQDG (ES −1.31; CrI −2.60 to −0.06) in peanut, and LC-MS detected the increase in TG (ES 2.28; CrI 0.48 to 3.92) in Arabidopsis, consistent with pooled analysis, while none of the platforms detected the changes in PE (Supplementary Table S11). Some lipid alterations detected by specific lipid-profiling platforms were not detected by pooled analysis across platforms. For example, DI-MS/MS and LC-MS detected a decrease in PG in Arabidopsis (ES −1.01; CrI −2.03 to −0.20 under DI-MS/MS, and ES −2.70; CrI −4.90 to −0.15 under LC-MS); DI-MS/MS detected a decrease in PA (ES −0.78; CrI −1.78 to −0.02) in soybean; and LC-MS/MS detected an increase in DG (ES 1.22; CrI 0.08 to 2.42) in Arabidopsis under heat stress across all tissues and growth stages. When we checked the tissue-specific lipid alterations under heat stress for each lipid-profiling platform separately, we found that DI-MS/MS detected the decrease in PC (ES −0.60; CrI −1.11 to −0.08) in leaves, consistent with pooled analysis, across all plant species and growth stages (Supplementary Table S12). None of the platforms detected differences in PA, PG, or SQDG levels in any tissues. The detailed responses for each plant species and tissue type within each lipid-profiling platform are given in Supplementary Table S13. The split-platform sensitivity analysis of the lipid class responses (which considers only the directional consistency of the estimates, ignoring statistical significance of changes) found that directional consistency was 60% across the three lipid-profiling platforms (∼0.6 sign match), with Pearson correlation coefficients ranging from 0.3 to 0.4 (Supplementary Table S14).

**Fig. 5.**
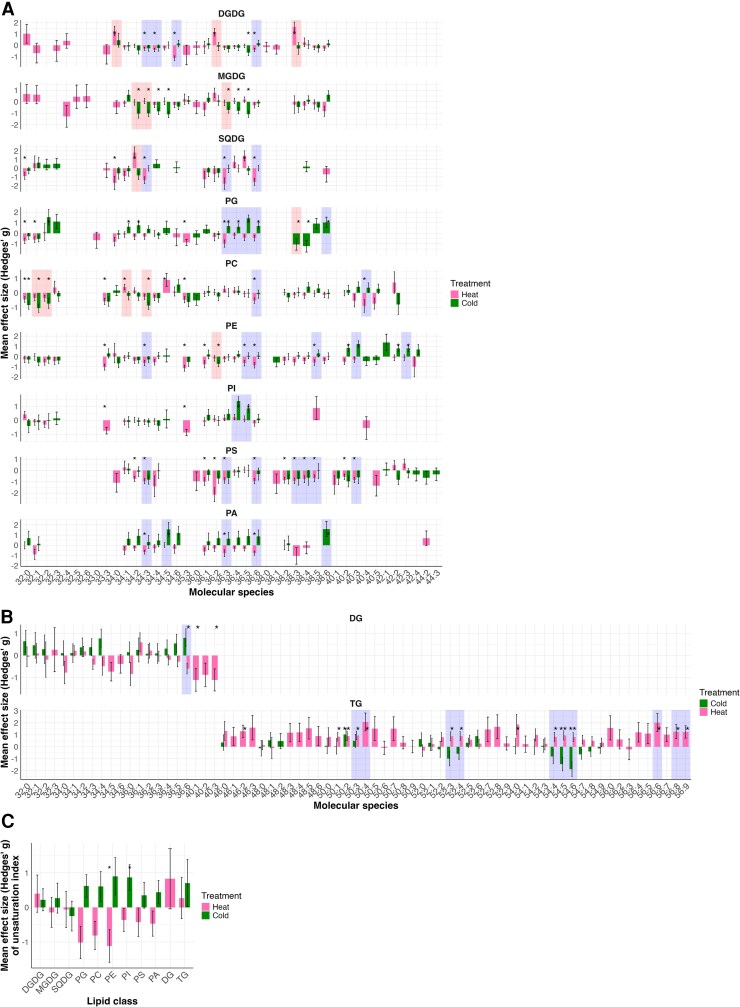
Alterations in the levels of lipid molecular species (total number of carbons:total number of double bonds) of the lipid classes. (A) Digalactosyldiacylglycerol (DGDG), monogalactosyldiacylglycerol (MGDG), sulfoquinovosyldiacylglycerol (SQDG), phosphatidylglycerol (PG), phosphatidylcholine (PC), phosphatidylethanolamine (PE), phosphatidylinositol (PI), phosphatidylserine (PS), and phosphatidic acid (PA); (B) diacylglycerol (DG) and triacylglycerol (TG), and (C) unsaturation indices of all the major lipid classes under heat and cold stresses. An asterisk indicates a significant change determined with 95% credible interval. The blue rectangles indicate the significant changes in highly unsaturated lipid species (e.g. decreased under heat stress and increased under cold stress for diacyl lipids in A) and the orange rectangles indicate the significant changes in less unsaturated and saturated lipid species (e.g. increased under heat stress and decreased under cold stress for diacyl lipids in A). (C) Indicates how the above changes led to an overall decrease in the unsaturation indices of most lipid classes under heat stress and an increase in the unsaturation indices of most lipid classes under cold stress.

The pooled meta-analysis revealed that the highly unsaturated lipid species (DGDG 34:3, 34:4, 34:6, and 36:6; SQDG 34:3, 36:3, and 36:6; DG 36:6; PA 34:3, 36:3, and 36:6; PG 36:3; PC 36:6 and 40:4; PE 34:3, 36:5, 36:6, and 38:5; and PS 34:3, 36:3, 36:6, 38:3, 38:4, 38:5, and 40:3) decreased under heat stress. In contrast, the less unsaturated lipid species and saturated lipid species increased (DGDG 34:0, 36:2, and 38:3; SQDG 34:2; PC 34:1) under heat stress across plant species, tissue types, and growth stages (Fig. 5A, B). The unsaturation index of PE decreased under heat stress (Fig. 5C). A contrasting trend was observed with TG: highly unsaturated species (50:3, 50:4, 52:3, 52:4, 54:4, 54:5, 54:6, 56:6, 56:8, and 56:9) increased under heat stress. The lipid changes described above were similar across species, tissue types, and growth stages. Under the split-platform analysis, DI-MS/MS detected the decrease in highly unsaturated lipid species; DG 36:6; PA 34:3, 36:3, and 36:6; PC 36:6 and 40:4; PE 34:3, 36:5, 36:6, and 38:5; PG 36:3; and PS 34:3, 36:3, 36:6, 38:3, and 40:3 under heat stress (Supplementary Fig. S4), consistent with pooled analysis. DI-MS/MS also detected the increase in the less unsaturated lipid species DGDG 38:3 and PC 34:1 under heat stress across plant species, tissue types, and growth stages (Supplementary Fig. S4A, B), similar to the pooled analysis. Further, DI-MS/MS detected lipid alterations that were not revealed through the pooled analysis. For example, DI-MS/MS detected decreases in DG 40:3, MGDG 38:6, PC 34:3 and 34:4, PE 34:4 and 38:3, PG 36:5 and 36:6, and PI 34:3 and 36:6, and increases in MGDG 36:2 and PC 34:2 and 36:3 under heat stress, which were not revealed through the pooled analysis. Changes that the pooled analysis detected and DI-MS/MS failed to detect included decreases in DGDG 34:3, 34:4, 34:6, and 36:6, SQDG 34:3, 36:3, and 36:6, and PS 38:4 and 38:5, and increases in DGDG 34:0 and 36:2, and SQDG 34:2. Consequently, the unsaturation indices of PC, PE, and PI decreased under heat stress with DI-MS/MS-specific data (Supplementary Fig. S4C). DI-MS/MS also detected the increase in highly unsaturated TG (50:3, 52:3, 52:4, 54:4, and 54:5) under heat stress. LC-MS/MS detected several lipid species changes that DI-MS/MS and/or pooled analysis detected (Supplementary Fig. S5). LC-MS/MS also detected lipid alterations not revealed through the other two (Supplementary Fig. S5); for example, LC-MS/MS detected a decrease in MGDG 34:6 and PC 36:4 and an increase in TG species 54:8 under heat stress.

**Fig. 6.**
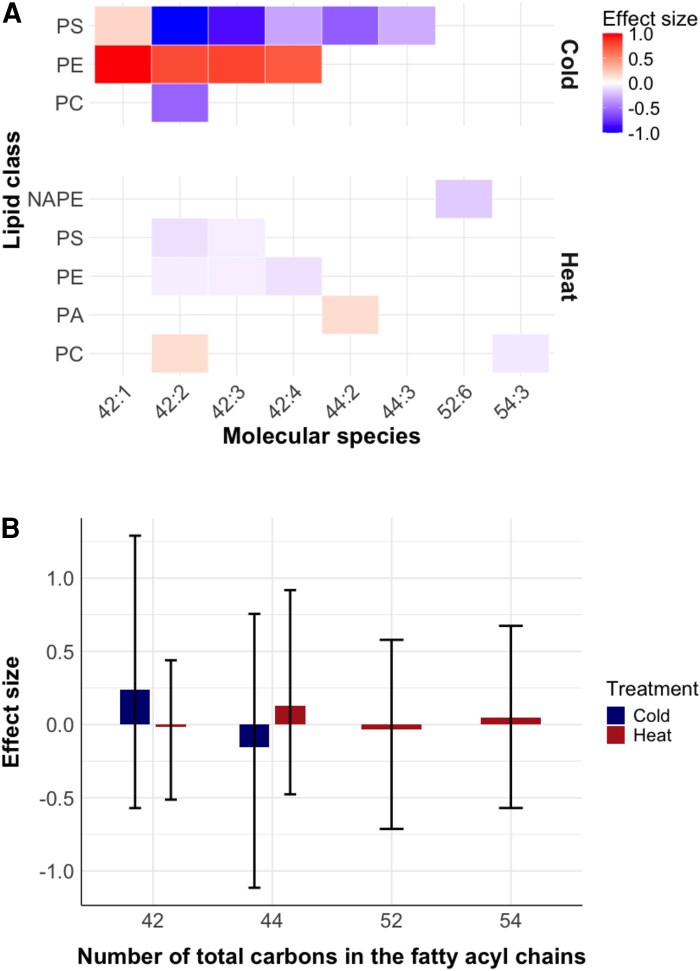
Response of very-long-chain fatty acid (≥C22)-containing lipids under heat stress and cold stress. The effect size refers to the posterior estimate of the standardized mean difference between the temperature treatment and control under each stress, calculated as Hedges’ g. In (A), a positive value for effect size (highlighted in red shade) indicates an increase in the corresponding lipid, and a negative value (highlighted in blue shade) indicates a decrease. In (B), bars represent the mean values, and the black lines indicate 95% credible intervals.

A total of 15 studies reported data on the alterations in the levels of VLCFAs (number of carbon atoms ≥22 in a single acyl chain) under both heat and cold stresses. A consistent change in the levels of VLCFAs was not observed under heat stress (Fig. 6). While PS and PE lipids with C42 acyl chains showed a slight decrease under heat stress, PC with C42 acyl chains showed an increase; however, none of these changes was statistically significant. Similarly, C44 PA increased and C54 PC decreased, but the changes were not significant.

### The effect of cold stress on the plant lipidome

When the temperature drops, plants alter their membrane lipid composition to maintain membrane flexibility in a manner opposite to heat stress. Saturated fatty acids pack together with more contact points than unsaturated fatty acids. Therefore, replacing saturated fatty acids with unsaturated at varying extents or invoking desaturase activity to increase the degree of unsaturation increases membrane fluidity (Moellering *et al*., 2010; Chen and Thelen, 2013). In addition, the stability of chloroplast membranes relies on partially replacing head groups that change the ratio of DGDG to MGDG. Consistently, in our work, significant remodeling of the lipidome was observed in the tested plant species under cold stress (Fig. 3B; Supplementary Table S5). Based on the pooled meta-analysis, conducted across lipid-profiling platforms, levels of PA (ES 1.10; CrI 0.29 to 1.85), lysophosphatidylethanolamine (LPE; ES 1.33; CrI 0.35 to 2.21), and DG (ES 1.17; CrI 0.15 to 2.19) increased under cold stress across all plant species, tissue types, and growth stages (Supplementary Table S15). Some lipid alterations were plant species specific: for example, lysophosphatidylcholine (LPC) increased in peanut (ES 7.74; CrI 4.78 to 10.67) and Arabidopsis (ES 1.26; CrI 0.02 to 2.49); lysophosphatidylglycerol (LPG; ES −1.34; CrI −3.05 to −0.02) decreased in peanut; MGDG decreased in peanut (ES −1.02; CrI −2.05 to −0.18) and maize (ES −0.71; CrI −1.47 to −0.06); and DGDG (ES 1.27; CrI 0.21 to 2.28) increased in peanut across all tissue types and growth stages, while the levels of the same lipid classes did not change in other species under cold stress (Supplementary Table S16). When the lipid alterations were evaluated within the tissue types, an MGDG decrease (ES −0.61; CrI −1.18 to −0.05) was evident in leaves under cold stress across all plant species and growth stages, while the same trend was not significant for other tissues (Supplementary Table S17). The detailed responses for each plant species and tissue type are presented in Fig. 4B and Supplementary Table S18.

The split-platform analysis (i.e. data were analyzed on DI-MS/MS, LC-MS/MS, and TLC-GC platforms separately) revealed results that were both similar to and distinct from those of the pooled analysis. For example, DI-MS/MS detected the increase in PA (ES 1.88; CrI 0.19 to 2.99) and LC-MS/MS detected the increase in LPE (ES 1.66; CrI 0.03 to 3.06) across all plant species, tissue types, and growth stages, similar to the pooled analysis (Supplementary Table S19). However, LC-MS/MS and TLC-GC did not detect the changes in PA levels, and DI-MS/MS and TLC-GC did not detect the differences in LPE levels under cold stress, while none of the platforms detected any changes in DG levels. Additionally, LC-MS/MS detected a decrease in MGDG (ES −0.79; CrI −1.45 to −0.12) across all plant species, tissues, and growth stages, which was not detected by DI-MS/MS, TLC-GC, and the pooled analysis. When plant species-specific alterations were checked with the lipid-profiling platforms, we found that LC-MS/MS detected the increase in LPC (ES 8.99; CrI 6.07 to 11.92) and decrease in LPG (ES −1.59; CrI −3.50 to −0.19) in peanut, consistent with the pooled analysis (Supplementary Table S20). When we checked the tissue-specific lipid alterations under cold stress for each lipid-profiling platform separately, we found that LC-MS/MS detected the decrease in MGDG (ES −0.94; CrI −1.64 to −0.21) and increase in DG (ES 1.41; CrI 0.02 to 2.65) in leaves, consistent with the pooled analysis, across all plant species and growth stages (Supplementary Table S21). The detailed responses for each plant species and tissue type within each lipid-profiling platform are given in Supplementary Table S22. The split-platform sensitivity analysis found that directional consistency was >85% between DI-MS/MS and LC-MS/MS (∼0.88 sign match) with a Pearson correlation coefficient of 0.4, whereas TLC-GC and the other two platforms showed only 30% consistency (∼0.33 sign match) (Supplementary Table S14).

The pooled meta-analysis revealed that the highly unsaturated lipid species (PA 34:5 and 38:6; PG 36:3, 36:4, 36:5, 36:6, and 38:6; PE 40:3 and 42:3; PI 36:4 and 36:5) increased under cold stress. In contrast, the less unsaturated lipid species (MGDG 34:2, 34:3, and 36:3; PG 38:3; PC 32:1, 32:2, and 34:3; and PE 36:2) and saturated lipids (PC 32:0) decreased under cold stress across plant species, tissue types, and growth stages (Fig. 5A, B). The unsaturation index of PI increased under cold stress (Fig. 5C). A contrasting trend was observed with TG: highly unsaturated species (54:5 and 54:6) decreased under cold stress. The lipid changes described above were similar across species, tissue types, and growth stages. Under the split-platform analysis, DI-MS/MS detected the increase in highly unsaturated lipid species (PG 36:5, PE 40:3, and PI 36:4) under cold stress (Supplementary Fig. S4), consistent with the pooled analysis. DI-MS/MS also detected the decrease in the less unsaturated lipid species and saturated lipid species (MGDG 34:3, PC 32:1 and 34:3, and PE 36:2) under cold stress across plant species, tissue types, and growth stages (Supplementary Fig. S4A, B), similar to the pooled analysis. Furthermore, DI-MS/MS detected lipid alterations that were not revealed by the pooled analysis across the platforms. For example, DI-MS/MS detected an increase in DGDG 34:6 and MGDG 38:6 and decreases in PC 34:1, 36:2, 36:3, and 38:2, PE 32:1, 32:3, 34:2, 34:3, and 36:3, and PG 32:0 and 32:1 under cold stress, which were not revealed through the pooled analysis. Changes that the pooled analysis detected and DI-MS/MS failed to detect included increases in PA 34:5 and 38:6, PG 36:3, 36:4, 36:6, and 38:6, PE 42:3, and PI 36:5, and decreases in MGDG 34:2 and 36:3, PG 38:3, and PC 32:0 and 32:2. DI-MS/MS did not detect the decrease in highly unsaturated TG species under cold stress. LC-MS/MS detected most lipid species changes that DI-MS/MS and/or the pooled analysis detected (Supplementary Fig. S5). LC-MS/MS also detected some lipid alterations that were not revealed through the other two (Supplementary Fig. S5); for example, LC-MS/MS detected a decrease in PG 32:1 and TG 54:7, and an increase in PA 36:5 and 36:6, and PC 38:3 and 38:4 under cold stress. Responses of VLCFAs were rarely reported under cold stress. Overall, VLCFA-containing PE increased, and VLCFA-containing PC and PS decreased under cold stress (Fig. 6).

## Discussion

This systematic review summarizes 90 studies focusing on plant lipidome responses under temperature stress. The increase in studies examining lipidome responses to temperature extremes underscores the importance of lipid-remodeling mechanisms under fluctuating temperature conditions for climate-resilient crop production. Investigations on lipid responses under heat stress were more extensive and numerous compared with those under cold stress. The investigated plant species spanned a wide range, including the model species Arabidopsis, food, feed, and fiber crops, extremophytes, and wild relatives. Investigations primarily focused on the vegetative tissues (mainly leaves) rather than the reproductive tissues (anther, pollen, etc.). The temperature thresholds that define heat and cold stresses varied across studies, depending on plant species, tissue types, and growth stages; the findings presented here focus on robust plant lipidomic responses to heat and cold across temperature levels, plant species, tissue types, and growth stages.

The reviewed publications demonstrated the progression of lipid-profiling techniques over time. Early research typically measured lipids in broad categories such as phospholipids and glycolipids, or based on saturation levels, and the capabilities were enhanced with TLC and GC techniques (Novitskaya *et al*., 2000; Palacios *et al*., 2019). Advances in separation technologies were critical to the classification of lipid species, and a breakthrough occurred with the development of ESI. Fenn *et al*. (1989) revolutionized our ability to monitor small labile molecules with ESI, which is a ‘soft’ ionization technique that allows for the ionization of fragile polar molecules that play vital roles in biological systems. The adoption of ESI-MS/MS significantly improved sensitivity and precision in detecting various lipid components, enabling the detection of rare lipid molecules and their fatty acid chain length and unsaturation patterns. Moreover, tandem MS with ESI quantified compounds at picomole levels, making it possible to measure nearly 90% of lipid pools. As a result, ESI-MS/MS has become the dominant platform in lipidomics, offering sensitivity several orders of magnitude higher than that of previous methods, and holds much promise with isotopes for stable-isotope-based metabolic flux analyses of lipid pathways (Allen, 2016). These advances have greatly strengthened our ability to understand lipid metabolic networks and assess lipidome alterations in plant stress responses (Allen *et al*., 2015; Wang *et al*., 2025).

The major responses of the lipidome to temperature fluctuations were head group changes, alterations in unsaturation levels of the membrane lipids, and sequestering the fatty acyl chains to neutral lipid buffers. These interconnected lipid-remodeling mechanisms affect membrane fluidity and stability. Under heat stress conditions, a major head group change across plant species and tissue types was a decrease in MGDG, indicating a possible adaptation to prevent the formation of a non-bilayer phase. Due to the presence of a single sugar group attached to the glycerol backbone, MGDG forms a cone-shaped structure that can make the membrane leaky (Li-Beisson *et al*., 2013). Hence, a decrease in MGDG can reduce the propensity of membranes to undergo phase transitions under heat stress. It has been reported that MGDG synthase 1 (MGD1), which transfers a galactose from UDP-galactose to DG and produces MGDG, is down-regulated under heat stress (Higashi *et al*., 2015; Srirangan *et al*., 2015). Further, Arabidopsis exhibited a decrease in PE, another non-bilayer-forming lipid. These compositional alterations probably counteract the excessive membrane fluidity and potential transitions to non-bilayer phases at high temperature. Additionally, a prominent change observed under heat stress was the elevated levels of TG across all plant species, tissues, and growth stages (Fig. 4). This increase in TG under heat stress might be related to an up-regulation of the Kennedy pathway that facilitates *de novo* synthesis of TG.

Under cold stress, an increased DGDG–MGDG contrast (differences in the estimated effect sizes of DGDG and MGDG, a measure to reflect the relative abundance of DGDG and MGDG) was detected (ES 0.63; CrI 0.36 to 0.93), which might be an adaptation to prevent destabilization of membranes due to cell dehydration that can occur under severe cold/freezing injury (Steponkus *et al*., 1990). Low temperatures (around –4 °C to −10 °C) can lead to lamellar-to-hexagonal phase II transitions in plasma membrane and chloroplast envelope membranes due to dehydration of membrane lipids (Welti *et al*., 2002). Lipids with larger head groups, such as DGDG, form bilayers while lipids with smaller head groups, such as MGDG, tend to form non-bilayer phases (Graham *et al*., 1973; Cullis and De Kruijff, 1979; Seddon, 1990; Jouhet, 2013; Marsh, 2013). Higher ratios of DGDG to MGDG help plants to prevent membrane transition to non-bilayer phases and maintain membrane stability under severe cold stress (Webb and Green, 1991; Higashi and Saito, 2019). Additionally, increases in PA, DG, and lysophospholipids were also observed under cold stress. Corresponding with an increase in DG, there was an associated decrease in MGDG and DGDG containing acyl chains with the same number of carbon atoms (e.g. DG 36:4 increased and DGDG 36:2 and MGDG 36:2 decreased under cold stress). This lipid remodeling involving chloroplast lipids could potentially be associated with the activity of the galactolipid:galactolipid galactosyltransferase (GGGT) enzyme (Moellering and Benning, 2011). Under severe cold/freezing stress, GGGT transfers a galactosyl moiety from MGDG onto another galactoglycerolipid, such as MGDG or DGDG, producing oligogalactodiacylglycerols [e.g. DGDG, trigalactosyldiacylglycerol (TGDG), and tetragalactosyldiacylglycerol (TeDG)] and releasing DG as a by-product (Moellering and Benning, 2011). The GGGT mechanism helps prevent freeze-induced cellular dehydration and stabilizes chloroplast envelope membranes. It has been reported that most of the PA accumulated under cold stress has been generated via the sequential action of phospholipase C (PLC) and diacylglycerol kinase (DGK), and ∼20% of PA has been generated via phospholipase D (PLD) (reviewed by Testerink and Munnik, 2005). PLC hydrolyzes phosphatidylinositol-4,5-bisphosphate (PIP_2_) into inositol-14,5-trisphosphate and DG, which is rapidly phosphorylated to PA by DGK. PLD hydrolyzes structural phospholipids to generate PA and a free head group. PA is an important signaling molecule under stress, and it plays key roles in plant development and hormonal responses (Chen *et al*., 2010; Yao and Xue, 2018; Wu *et al*., 2022). PA-binding proteins are also key in lipid metabolism (Dubots *et al*., 2010; Yao and Xue, 2018). However, the PA accumulation in response to freezing might be caused partially by cell injury, which activates PLDα (Wang *et al*., 2000). Different PLD isoforms have different effects on cold response [increased cold tolerance via PLDα deficiency (Welti *et al*., 2002; Welti and Wang, 2004) or decreased cold tolerance with PLDδ deficiency (Li *et al*., 2004)]. These data suggest that accumulation of PA can either reflect lipid turnover indicative of cold damage or be part of an adaptive response to equip the plant against low temperatures (Zhao *et al*., 2021). It appears that the outcome of PA accumulation depends upon the PA species; for example, PA 34:6 accumulation is associated with freezing tolerance, while the accumulation of most other PA species is associated with susceptibility (Testerink and Munnik, 2005; Vu *et al*., 2022). Increased synthesis of PA might have led to an increased synthesis of DG under cold stress through the activity of PA phosphatases (PAPs) (Meijer and Munnik, 2003; Zhang *et al*., 2020; Sharma *et al*., 2023). Further, while their precise biological roles are not fully resolved, lysolipids appear to be recurring features of the cold-stressed lipidome and warrant deeper investigation (Lee and Ridgway, 2020; Liu *et al*., 2022; Sharma *et al*., 2023; Zhang *et al*., 2023; Gao *et al*., 2024). Accumulated LPE and LPC might reflect increased hydrolysis of PE and PC, respectively, that could be facilitated by phospholipase A (PLA) (Bates, 2016).

Our meta-analysis showed that altering the unsaturation levels of membrane lipids to maintain optimal fluidity and stability of membranes is a robust adaptation mechanism of plant lipidomes to temperature stresses. Under heat stress, a broad shift from highly unsaturated lipids to less unsaturated and saturated lipids was observed as evidenced across plastidic and extraplastidic membrane lipid classes, lipids with polyunsaturated fatty acids (e.g. 18:3, linolenic acid) declined, while lipids with less unsaturated fatty acids (e.g. 18:1, oleic acid; 18:2, linoleic acid) or saturated fatty acids (16:0, palmitic acid) increased under heat stress. As a result, unsaturation indices of lipid classes decreased (Fig. 5). Fatty-acyl chains in plant membranes commonly have *cis* double bonds that reduce the degree of compaction in membranes by introducing bends in the acyl chains (Huang, 2006). High temperatures also decrease membrane compactness. By decreasing the number of double bonds under high temperatures, plants can maintain optimal membrane compactness and, consequently, optimal membrane fluidity and integrity. The alterations in the membrane lipid unsaturation levels followed an opposite trend under cold stress (highly unsaturated acyl chains increased and less unsaturated and saturated acyl chains decreased) as the purpose was to counteract the decrease in membrane fluidity and transition to non-bilayer gel phase under low temperatures. Recent studies also supported the acyl sequestration mechanism between the membrane lipids and the neutral lipid buffers (Légeret *et al*., 2016; Spivey *et al*., 2023). The acyl sequestration was manifested predominantly by TGs that took up the acyl chains expelled from the membrane lipids. For example, under heat stress, the 18:3 acyl chains from membrane lipids were incorporated into TGs, which decreased the unsaturation levels of membrane lipids, as reported by Narayanan *et al*. (2016a), Sun *et al*. (2022), and Bian *et al*. (2023). In Arabidopsis, a soluble diacylglycerol acyltransferase (DGAT3) catalyzes the incorporation of 18:3 fatty acids into TGs in an acyl-CoA-dependent pathway of TG biosynthesis (Hernández *et al*., 2012). In an acyl-CoA-independent pathway, phospholipid:diacylglycerol acyltransferase (PDAT) catalyzes the transfer of an acyl group from the *sn*-2 position of a phospholipid, mainly PC, to the *sn*-3 position of DG, giving rise to TG and a lysophospholipid (Dahlqvist *et al*., 2000; Zhang *et al*., 2022). Higashi and Saito (2019) reported the overexpression of both PDAT and DGAT under heat stress. It has been reported that plants may direct the free fatty acids to TG under environmental stresses to increase the energy reserve and prevent cellular damage caused by free fatty acids (known as lipotoxicity) (Xu and Shanklin, 2016; Mueller *et al*., 2017; Bates, 2022; Henschel *et al*., 2024). Further, the decreases in the levels of 18:3-containing MGDG species (such as 36:6 and 38:6) under heat stress (Fig. 5A) could be related to the action of heat-inducible lipase 1 (HIL-1) that hydrolyzes MGDG and removes polyunsaturated fatty acids (Higashi *et al*., 2018; Kan *et al*., 2023). These fatty acids released from MGDG may travel through PC to reach TG (Karki *et al*., 2019), where the final conversion from DG to TG is supported by PDAT and DGAT (Mueller *et al*., 2017; Shiva *et al*., 2020). Collectively, these reports support the role of TGs in plant adaptation to heat stress.

Apart from lipidomics, other MS measurements indicate the presence of polyunsaturated acyl-ACPs (i.e., 16:3-ACP) in plant tissues (Nam *et al*., 2020). Because polyunsaturated acyl-ACPs are not part of classical fatty acid biosynthesis, acyl-activating synthetases (Koo *et al*., 2005; Tjellström *et al*., 2013) may play a more important role than previously thought in acyl chain partitioning and recycling within the chloroplast under ambient or stressed temperature conditions. However, studies under altered temperature conditions have only begun to investigate the effects on acyl-ACP pools (Jaroensuk *et al*., 2026). As acyl-ACP measurements are non-trivial (Jenkins *et al*., 2021), these additional aspects of lipid metabolism are not considered in current lipidomic experiments, but will continue to shape our evolving mechanistic understanding.

Overall, the meta-analysis results indicate that plants use an acyl-recycling mechanism to adapt to temperature stress, with TG frequently serving as a transient ‘warehouse’ for cleaved fatty acids. There is now evidence for active remodeling of TG, often involving unusual or unanticipated fatty acids (Parchuri *et al*., 2024) and this probably occurs under stress. Furthermore, beta oxidation of fatty acids occurs in leaves and developing seeds (Graham and Eastmond, 2002; Goepfert and Poirier, 2007) with recent supporting evidence from stable isotope (Koley *et al*., 2025) and radiolabeling (Johnson *et al*., 2025) investigations. These studies indicate that our mechanistic understanding of fatty acid partitioning remains incomplete and represents an active area for future studies on temperature effects to complement lipidomics.

Studies have also proposed a potential role for VLCFAs in plant stress adaptation, yet systematic evidence remains limited due to their under-representation in lipidomic studies (De Bigault Du Granrut and Cacas, 2016). The extended hydrocarbon chains of VLCFAs increase van der Waals interactions among lipid molecules, potentially enhancing membrane packing and preventing membrane destabilization under temperature stress (Lynch and Dunn, 2004). However, our meta-analysis did not reveal conclusive evidence of the role of VLCFAs in heat or cold stress adaptation (Fig. 6). A consistent increase or decrease of VLCFAs was not observed under heat or cold stress. This may partly be due to the limited number of studies that have reported VLCFAs. As our study highlights, a deeper understanding of VLCFA dynamics across lipid classes, tissues, and developmental stages is essential to elucidate their precise role in plant stress adaptation.

**Fig. 7.**
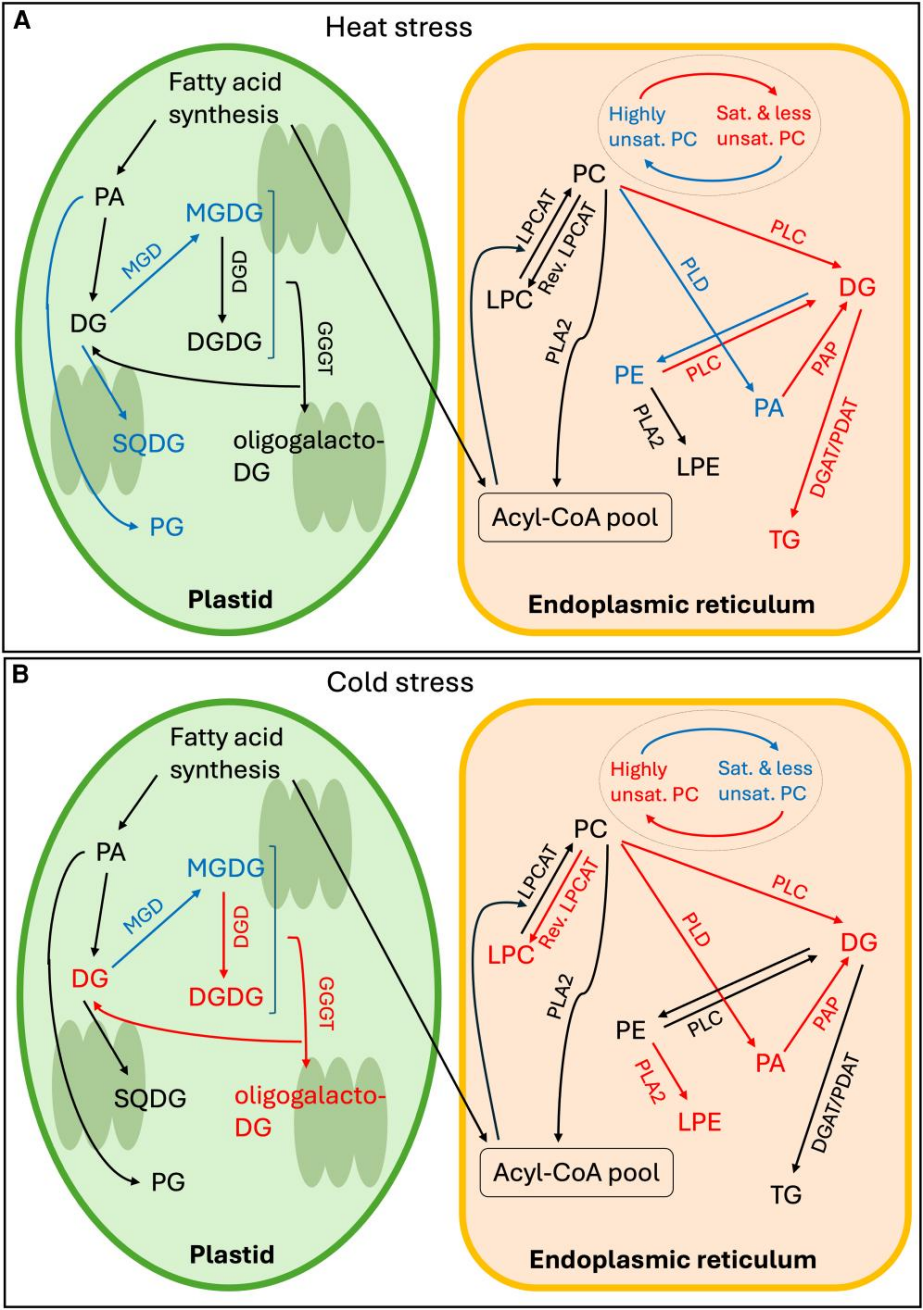
The lipid remodeling of plants under heat and cold stress. Lipid remodeling under heat stress (A) and cold stress (B), revealed through the meta-analysis and systematic review. The lipids, enzymes, and reactions (arrows) highlighted in red and blue represent an increase and decrease, respectively, under stress. MGDG, monogalactosyldiacylglycerol; DGDG, diacylgalactosyldiacylglycerol; SQDG, sulfoquinovosyldiacylglycerol; PG, phosphatidylglycerol; PC phosphatidylcholine; PE, phosphatidylethanolamine; PA, phosphatidic acid; LPC, lysophosphatidylcholine; LPE, lysophosphatidylethanolamine; DG, diacylglycerols; TG, triacylglycerol; MGD, MGDG synthase; DGD, DGDG synthase; GGGT, galactolipid:galactolipid galactosyltransferase; LPCAT, lysophosphatidylcholine acyltransferase; PLA2, phospholipase A2; PLC, phospholipase C; PLD, phospholipase D; PAP, phosphatidic acid phosphatase; DGAT, diacylglycerol acyltransferase; and PDAT, phospholipid:diacylglycerol acyltransferase.

The lipid-remodeling pathway under heat and cold stresses, as revealed by this meta-analysis and systematic review, is summarized in Fig. 7 which places it in the broader context of plant lipid metabolism. As depicted in the figure, fatty acids synthesized in the plastid are incorporated into plastidic lipids or exported to the endoplasmic reticulum (ER) and incorporated into extra-plastidic lipids with or without further modifications. The fatty acids synthesized in the plastid are subsequently incorporated into lipid precursors, such as PA and DG (reviewed by Ohlrogge and Browse, 1995). Plastidic lipids MGDG, DGDG, and SQDG are synthesized from DG, which is mostly generated from the dephosphorylation of PA by PAPs. MGDG synthase (MGD) transfers a galactose molecule from UDP-galactose to DG, forming MGDG (probably decreases under heat and cold stresses) (Higashi *et al*., 2015; Srirangan *et al*., 2015). Further, DGDG synthase (DGD) transfers a galactose molecule from UDPGal to MGDG, forming DGDG (probably increases under cold stress). The enzyme GGGT transfers a galactosyl group from MGDG onto another MGDG or DGDG, thus producing DGDG or higher oligogalactodiacylglycerols, and releasing DG as a by-product (Moellering and Benning, 2011). This process appeared to increase under cold stress. PA can be used for the synthesis of PG, and DG can be used for the synthesis of SQDG in the plastids, and these processes appear to decrease under heat stress in some plant species. Fatty acids undergo desaturation through the action of fatty acid desaturases (FADs) in the chloroplast and ER. Fatty acids can be hydrolyzed from the plastidic and extra-plastidic lipids by various acyl hydrolases (Grienenberger *et al*., 2010; Higashi *et al*., 2018). In the ER, lysophosphatidylcholineacyltransferase (LPCAT) incorporates acyl-CoA into LPC and produces PC (Karki *et al*., 2019). Fatty acids attached to PC can return to the acyl-CoA pool via the reverse reaction of LPCAT or via PLA2; be transferred to DG by shuffling of acyl groups by CDP-choline:diacylglycerol choline phosphotransferase (DAG-CPT) or phosphatidylcholine:diacylglycerol cholinephosphotransferase (PDCT), removal of the entire phosphoryl head group via PLC, removal of the PC headgroup by PLD producing PA followed by removal of the remaining phosphate by PAP, or incorporation of modified fatty acids from the acyl-CoA pool into DG through the Kennedy pathway; or be transferred to PE lipids by first converting to DG lipids as previously mentioned followed by transfer of an ethanolamine head-group by CDP-ethanolamine:diacylglycerol cholinephosphotransferase (DAG-EPT) (Hu *et al*., 2012; reviewed by Bates, 2016). The enzyme PLA2 hydrolyzes PE into LPE as well as PC into LPC (Bates, 2016); this process is likely to be increased under cold stress. DG can be incorporated into TG along with fatty acids via PDAT or DGAT (Dahlqvist *et al*., 2000; Schnurr *et al*., 2002; Hernández *et al*., 2012), and this process is increased under heat stress.

This review identified critical knowledge gaps regarding plant lipidome responses to temperature stress. Lipid classes, such as sphingolipids and sterols, are characterized poorly. These lipid classes are involved in the structure of membrane microdomains, programmed cell death, and oxidative signaling (Gulati *et al*., 2010; Berkey *et al*., 2012). Including them in future analyses could reveal new regulatory mechanisms and stress markers. Moreover, the lipids that are oxidized and acylated were inconsistently grouped and under-reported across publications. Only a few studies distinguished and reported acylated lipids, while the other studies broadly grouped them under their respective headgroup class. Further, a strong bias toward model species such as Arabidopsis, rice, maize, and wheat, and vegetative tissues (mainly leaves) limits our understanding of how lipid remodeling contributes to reproductive success and yield stability under stress across different plant species. Additionally, variability in methodologies, including lipid extraction and profiling techniques, hampers cross-study comparisons. A meta-analysis conducted across pooled data generated through different lipid-profiling platforms (e.g. DI-MS/MS, LC-MS/MS, LC-MS, and TLC-GC) may produce inaccurate conclusions due to inherent differences in lipid-profiling techniques. On the other hand, a current limitation of a split-platform meta-analysis (conducted within each lipid-profiling platform separately) is low statistical power due to the limited number of studies within platforms. To address these limitations, we conducted a ‘pooled’ and ‘split-platform’ meta-analysis and provided a comparison of results. Additionally, we compared the directional consistency and correlation of the results across the individual platforms. Our work also reveals that the adoption of standard protocols and data reporting formats is critical, including a standardized format for naming lipid classes and molecular species. Community efforts to address such standardization could improve reproducibility and facilitate future meta-analysis. Finally, sparse data across various combinations of biological (e.g. plant species, tissues, and growth stages) and technical factors (e.g. lipid-profiling platforms and extraction protocols) can also limit the conclusions of a meta-analysis, which would potentially be overcome by more studies that might be conducted in the future.

## Conclusion

This meta-analysis underscores the critical role of lipid remodeling in plant adaptation to temperature stress. Our findings reveal conserved lipidomic responses to heat and cold stresses across plant species, tissue types, and growth stages. The decrease in the levels of lipids with relatively smaller head groups that promote membrane non-bilayers (e.g. MGDG), a decrease in unsaturation index in membrane lipids, and sequestration of polyunsaturated acyl chains into neutral lipid buffers (e.g. TG) emerge as conserved strategies for heat adaptation. This lipid remodeling probably counteracts the excessive membrane fluidity caused by high temperature and thus maintains membrane stability under heat stress. The alterations in membrane lipid head group composition and fatty acid unsaturation levels followed a different trend under cold stress (e.g. elevated polyunsaturated acyl chains and accumulation of PA). While the elevated polyunsaturated acyl chains are likely to reflect an adaptive shift favoring more fluid, flexible membranes, it is still not clear whether the accumulation of PA is a sign of damage or part of an adaptive response. Our analysis revealed key knowledge gaps in heat-adaptive lipid remodeling in reproductive tissues and the role of underexplored lipid classes, such as sphingolipids, sterols, and acylated and oxidized lipids, and VLCFAs in temperature stress adaptation. The analysis underscores the need for broader plant species coverage and methodological standardization in lipid profiling and reporting. Taken together, the current study provides a comprehensive understanding of lipidome remodeling in plants and its role in enhancing resilience to thermal stress, identifying priority areas for future research to support climate-resilient agriculture.

## Supplementary Material

erag183_Supplementary_Data

## Data Availability

All data are available in the article and its online supplementary data.

## Supplementary data

The following supplementary data are available at *JXB* online.

Fig. S1. The number of articles used in the systematic review as a function of year of publication.

Fig. S2. The results of the sensitivity analysis for heat stress and cold stress.

Fig. S3. The distribution of observed effect sizes of lipid molecular species among the 61 studies included in the meta-analysis.

Fig. S4. Lipid alterations detected by the DI-MS/MS lipid-profiling platform under heat and cold stresses.

Fig. S5. Lipid alterations detected by the LC-MS/MS lipid-profiling platform under heat and cold stresses.

Table S1. The metadata of all the studies included in the meta-analysis.

Table S2. Effect size calculated for lipid molecular species.

Table S3. Comparison of the fit of various meta models.

Table S4. Abbreviations of lipid classes.

Table S5. Results of the heterogeneity analysis (tests ‘between-study’ variability) of lipid classes under heat and cold stresses.

Table S6. The Bayesian effect size estimates for lipid classes from the pooled meta-analysis across lipid-profiling platforms under heat stress.

Table S7. The Bayesian effect size estimates for lipid classes of different plant species from the pooled meta-analysis across lipid-profiling platforms under heat stress.

Table S8. The Bayesian effect size estimates for lipid classes of different tissue types from the pooled meta-analysis across lipid-profiling platforms under heat stress.

Table S9. The Bayesian effect size estimates for lipid classes of different plant species and tissue types from the pooled meta-analysis across lipid-profiling platforms under heat stress.

Table S10. The Bayesian effect size estimates for lipid classes from the split-platform meta-analysis under heat stress.

Table S11. The Bayesian effect size estimates for lipid classes of different plant species from the split-platform meta-analysis under heat stress.

Table S12. The Bayesian effect size estimates for lipid classes of different tissue types from the split-platform meta-analysis under heat stress.

Table S13. The Bayesian effect size estimates for lipid classes of different plant species and tissue types from the split-platform meta-analysis under heat stress.

Table S14. The consistency of lipid-profiling platforms in terms of quantifying the lipid class responses to heat and cold stresses.

Table S15. The Bayesian effect size estimates for lipid classes from pooled meta-analysis across lipid-profiling platforms under cold stress.

Table S16. The Bayesian effect size estimates for lipid classes of different plant species from pooled meta-analysis across lipid-profiling platforms under cold stress.

Table S17. The Bayesian effect size estimates for lipid classes of different tissue types from pooled meta-analysis across lipid-profiling platforms under cold stress.

Table S18. The Bayesian effect size estimates for lipid classes of different plant species and tissue types from pooled meta-analysis across lipid-profiling platforms under cold stress.

Table S19. The Bayesian effect size estimates for lipid classes from the split-platform meta-analysis under cold stress.

Table S20. The Bayesian effect size estimates for lipid classes of different plant species from the split-platform meta-analysis under cold stress.

Table S21. The Bayesian effect size estimates for lipid classes of different tissue types from the split-platform meta-analysis under cold stress.

Table S22. The Bayesian effect size estimates for lipid classes of different plant species and tissue types from the split-platform meta-analysis under cold stress.
