# Supplementary material for: Conserved mechanisms of plant lipidome remodeling under heat and cold stresses revealed through a systematic review and meta-analysis
Source: J Exp Bot. 2026 Apr 17;77(11):3445–64. doi: 10.1093/jxb/erag183 (PMC13247527; doi:10.1093/jxb/erag183)
Supplement: erag183_Supplementary_Data [file erag183_supplementary_data.zip › jexbot316541-file001.pdf]

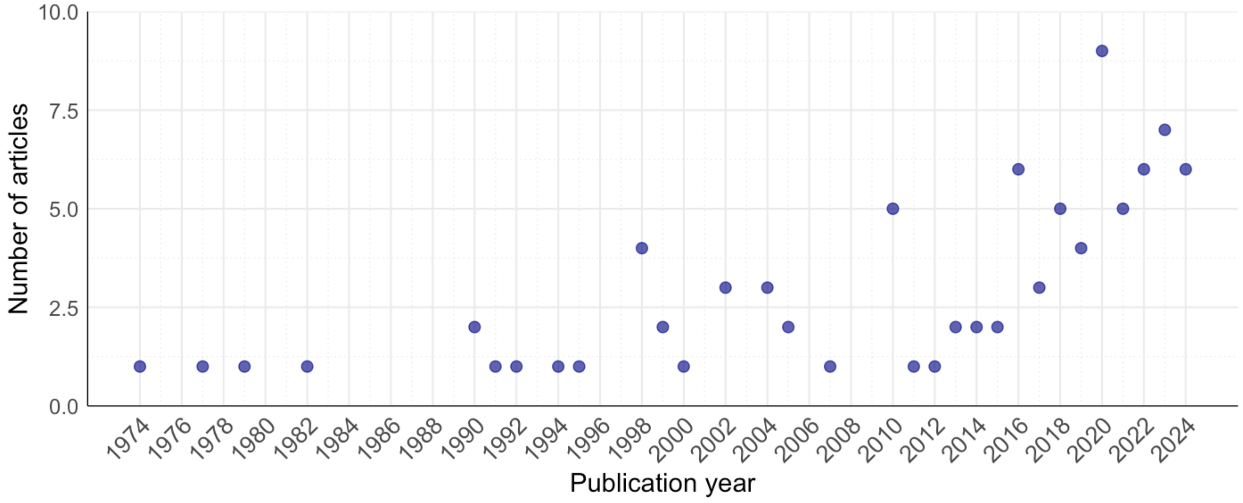


Figure S1: The number of articles as a function of year of publication for the 90 articles used in the systematic review.

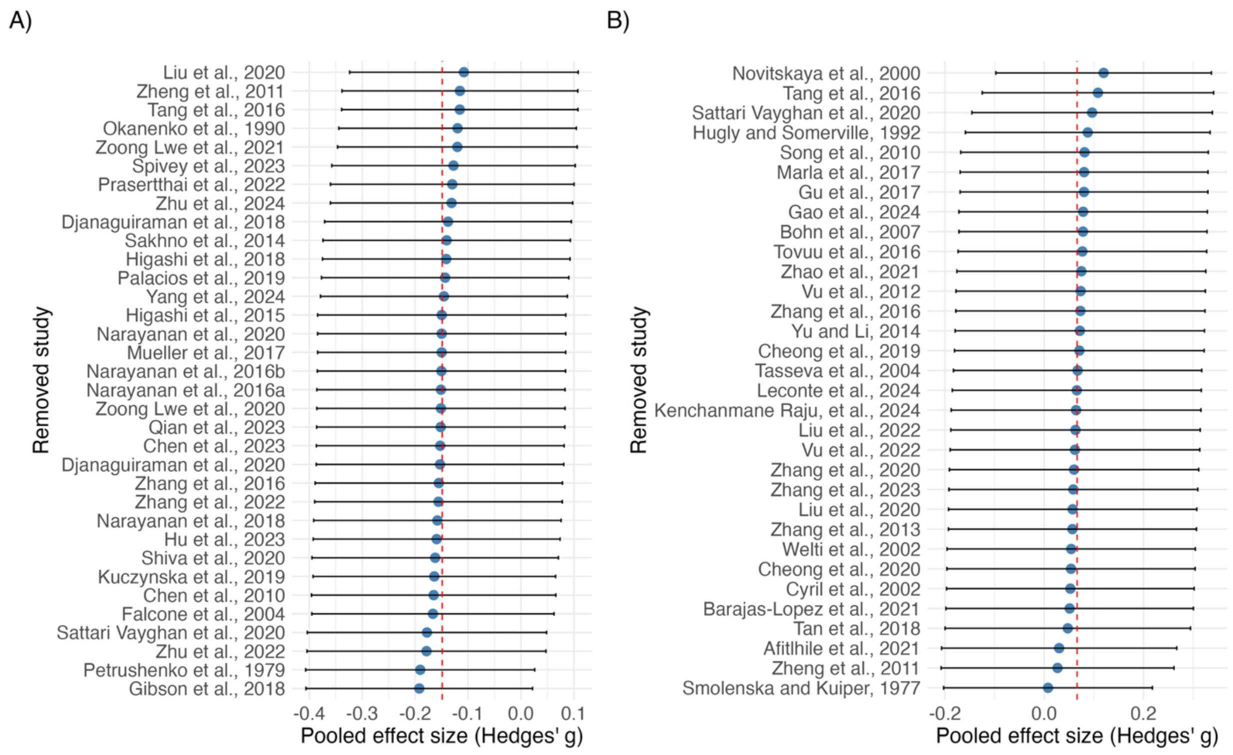


Figure S2: The results of the sensitivity analysis that checked whether any specific studies influenced pooled effect sizes in the meta-analysis done for heat stress (A) and cold stress (B). The sensitivity analysis removes one study at a time (presented on the Y-axis) and recalculates the overall effect size. The blue dots represent the pooled effect size (Hedges' g) recalculated after excluding the corresponding study on the Y-axis. The consistency of pooled effect sizes across all iterations (none of which deviated substantially from the overall effect estimate as indicated by the red line) showed that any single study didn't drive the overall conclusions, indicating that conclusions remained robust under heat and cold stresses.

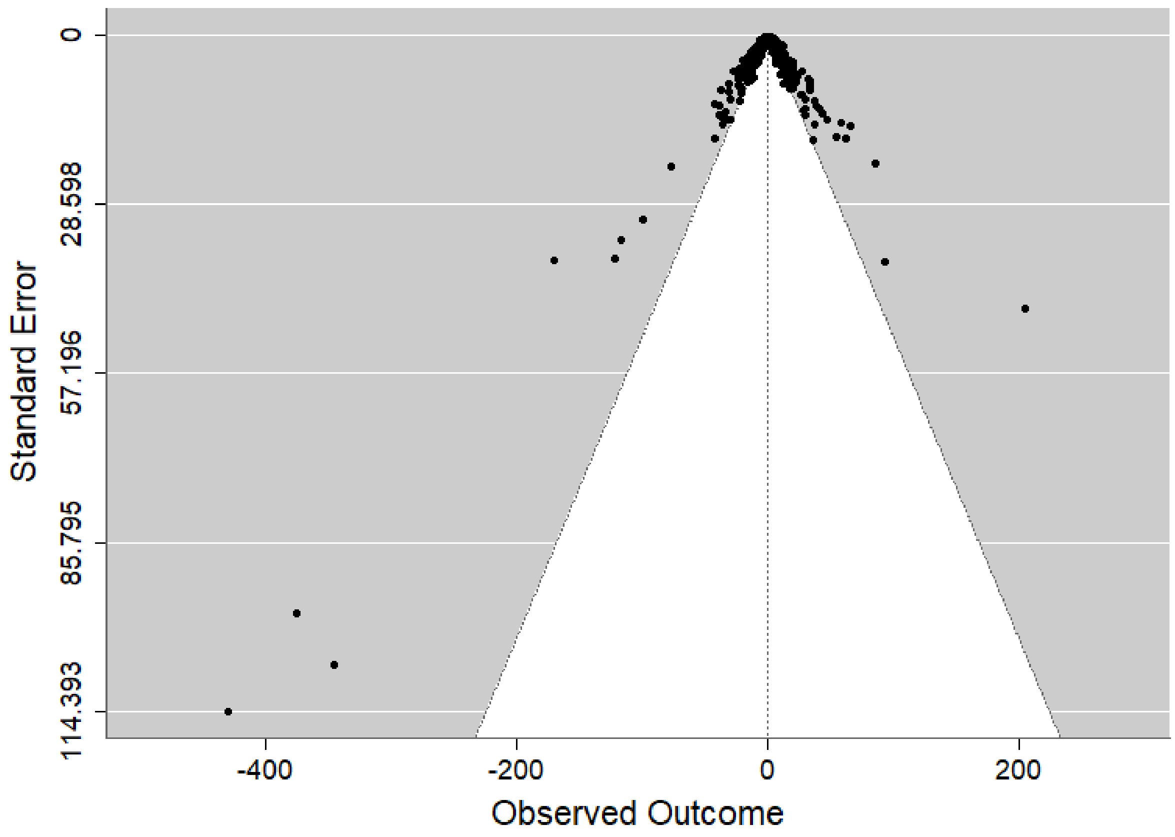


Figure S3: Funnel plot indicating the distribution of observed effect sizes of lipid molecular species among the 61 studies included in the meta-analysis. Each point represents a single study. The absence of asymmetry on either side of the inverted funnel suggests minimal evidence of publication bias (Egger's regression test, $p$ = 0.4874). In this plot, studies with smaller standard errors appear toward the top. The white region reflects the area outside the funnel's expected distribution. The grey-shaded region represents the expected 95% confidence limits around the pooled estimate, assuming no publication bias; studies outside this region (white region) may indicate potential outliers. The symmetry of points within the funnel shows that the meta-analytic estimate is unlikely to be skewed by selective reporting or small-study effects.

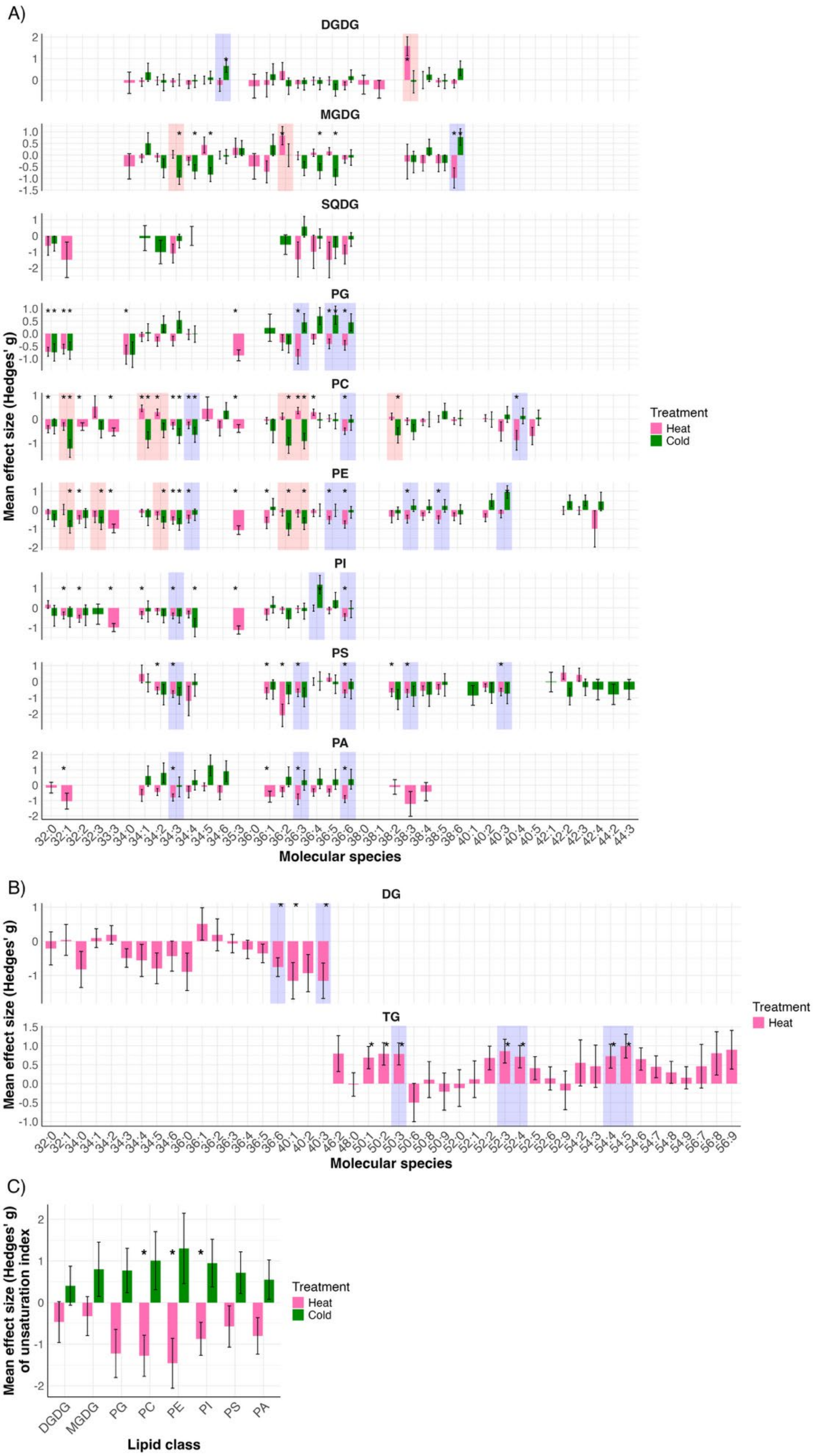
A)
DGDG
MGDG
SQDG
PG
PC
PE
PI
PS
PA
Mean effect size (Hedges' g)
Molecular species
Treatment
Heat
Cold
B)
DG
TG
Mean effect size (Hedges' g)
Molecular species
Treatment
Heat
C)
Mean effect size (Hedges' g) of unsaturation index
Lipid class
Treatment
Heat
Cold
DGDG
MGDG
PG
PC
PE
PI
PS
PA

Figure S4: Lipid alterations detected by the DI-MS/MS lipid-profiling platform under heat and cold stresses. An asterisk indicates a significant change determined with 95% credible interval. The blue rectangles indicate the significant changes in highly unsaturated lipid species (e.g., decreased under heat stress and increased under cold stress for diacyl lipids in Panel A) and the orange rectangles indicate the significant changes in less unsaturated and saturated lipid species (e.g., increased under heat stress and decreased under cold stress for diacyl lipids in Panel A). Panel C indicates how the above changes led to an overall decrease in the unsaturation indices of most lipid classes under heat stress and an increase in the unsaturation indices of most lipid classes under cold stress. DG, diacylglycerol; DGDG, digalactosyldiacylglycerol; MGDG, monogalactosyldiacylglycerol; PA, phosphatidic acid; PC, phosphatidylcholine; PE, phosphatidylethanolamine; PG, phosphatidylglycerol; PI, phosphatidylinositol; PS, phosphatidylserine; SQDG, sulfoquinovosyldiacylglycerol; TG, triacylglycerol.

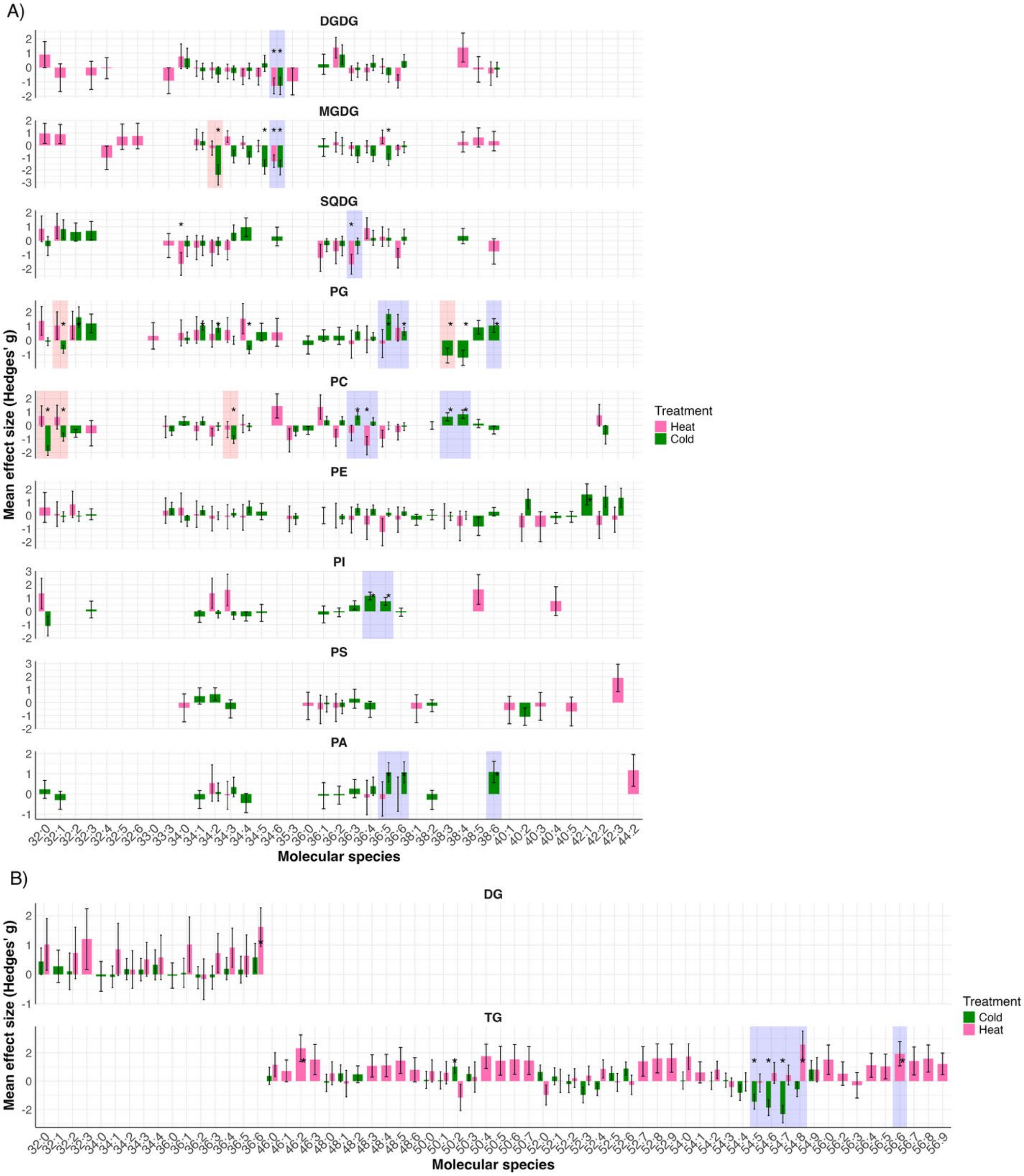


Figure S5: Lipid alterations detected by the LC-MS/MS lipid-profiling platform under heat and cold stresses. An asterisk indicates a significant change determined with 95% credible interval. The blue rectangles indicate the significant changes in highly unsaturated lipid species (e.g., decreased under heat stress and increased under cold stress for diacyl lipids in Panel A) and the orange rectangles indicate the significant changes in less unsaturated and saturated lipid species (e.g., increased under heat stress and decreased under cold stress for diacyl lipids in Panel A).

Since there were insufficient studies that reported data on the unsaturation indices under LC-MS/MS, the unsaturation indices plot is not presented here. DG, diacylglycerol; DGDG, digalactosyldiacylglycerol; MGDG, monogalactosyldiacylglycerol; PA, phosphatidic acid; PC, phosphatidylcholine; PE, phosphatidylethanolamine; PG, phosphatidylglycerol; PI, phosphatidylinositol; PS, phosphatidylserine; SQDG, sulfoquinovosyldiacylglycerol; TG, triacylglycerol.
